# The DNA mismatch repair protein, MSH6 is a novel regulator of PD-L1 expression

**DOI:** 10.1016/j.neo.2025.101207

**Published:** 2025-07-11

**Authors:** Kirsten Brooksbank, Charlotte Smith, Eleni Maniati, Amy Gibson, Wai Yiu Tse, Amy Kate Hall, Jun Wang, Tyson V Sharp, Sarah A Martin

**Affiliations:** a Centre for Cancer Cell & Molecular Biology, Barts Cancer Institute, Queen Mary University of London, Charterhouse Square, London EC1M 6BQ, UK; b Centre for Genomics & Computational Biology, Barts Cancer Institute, Queen Mary University of London, Charterhouse Square, London EC1M 6BQ, UK

**Keywords:** Mismatch repair, MSH6, Transcription factor, PD-L1, Immunotherapy

## Abstract

Immune checkpoint inhibitors (ICIs) are extremely effective in a subgroup of mismatch repair-deficient (MMRd) cancers, but ∼50% remain resistant to treatment. We have shown for the first time that this may be due to the differential regulation of factors linked to response to ICIs upon loss of the different MMR genes. Here, we show that increased PD-L1 expression is observed upon loss of the MMR genes MLH1, MSH2 and PMS2. However, this is not true upon loss of MSH6, and we show that this is due to a novel role for MSH6 as a direct regulator of PD-L1 transcription, dependent on recruitment by the histone trimethyltransferase SETD2. Next-generation sequencing of MLH1 and MSH6 knockout (KO) cells revealed that MSH6 KO cells have significantly lower microsatellite instability in comparison to MLH1 KO cells, despite MSH6 KO cells having a higher mutational burden. These findings emphasise the need for gene-specific stratification in the MMRd cohort.

## Introduction

The DNA mismatch repair (MMR) pathway is primarily concerned with the repair of DNA lesions that occur during DNA replication, such as base-base or insertion/deletion (indel) mismatches [1]. In humans, the MMR pathway is initiated by one of two different heterodimeric complexes of MutS-related proteins, MSH2-MSH6 (MutSα) or MSH2-MSH3 (MutSβ), each with a differing lesion recognition specificity. This interaction enables MutS to recruit the MutL complex, itself a heterodimer consisting of complexes of MLH1/PMS2 (MutLα) or MLH1/PMS1 (MutLβ), for the excision and repair of the damage. MMR-deficient (MMRd) tumours exhibit a mutator phenotype and are clinically characterized by high levels of microsatellite instability (MSI) [1]. This is a type of DNA damage that results in extensions and contractions in regions of the genome called microsatellites, which consist of short, repeated sections of nucleotides.

The use of immune checkpoint inhibitors (ICIs) has transformed the treatment of various solid tumours by blocking immunosuppressive signals to promote anti-tumour immunity. However, response rates are extremely variable necessitating the identification of predictive biomarkers for response. The most successful predictive biomarker for ICI response to date is MMRd/MSI-high (MSI-h), which received full approval for the treatment of metastatic or unresectable MSI-h/MMRd solid tumours with pembrolizumab in 2023 [[2], [3], [4]]. The high tumour mutational burden (TMB) in MMRd tumours is often credited with eliciting a strong immune response and, therefore, susceptibility to ICI treatment. However, resistance to anti-PD-1 therapy is observed in 40-70% of MMRd patients [5], highlighting the need to further refine our understanding of the mechanisms driving response and resistance to ICIs to better identify precise predictive biomarkers. There is accumulating evidence that MMRd/MSI-h is too broad a biomarker and that this could be why such variable response rates are observed in the MMRd/MSI-h cohort. For example, an *in vivo* study observed that MSH2 KO tumours with low MSI had a worse response to anti-PD-1 therapy than matched MSH2 KO tumours with high MSI [6]. In addition, genomic analysis from ICI-treated MMRd CRC and esophagogastric patient tumours also revealed that heterogeneity in MSI levels in the MMRd population may contribute to the variable response rates to ICIs observed. This is consistent with other studies that have shown that MMRd tumours are not necessarily MSI-h, even though MMRd/MSI-h received joint approval as a biomarker for response to pembrolizumab [7].

Another important factor associated with response to ICIs is expression of the immune checkpoint protein Programme Death Ligand 1 (PD-L1). Multiple anti-PD-1/PD-L1 antibodies, including pembrolizumab, target the PD-1/PD-L1 interaction and PD-L1 expression is utilised clinically to identify patients that may benefit from ICIs across several cancer types. PD-L1 expression is induced by both DNA damage and immune signalling, such as interferons (IFNs) and interleukins, via the JAK/STAT signalling axis [8,9]. Therefore, there is a logical link between MMRd/MSI-h and elevated TMB leading to increased PD-L1 expression, but there is conflicting evidence on whether this is the case. For example, numerous studies have demonstrated that PD-L1 expression and TMB are poorly correlated ([10]; Aurélien [11,12]). In addition, a retrospective analysis of MSI-h tumours found that TMB varies depending on whether MutS or MutL-related proteins had been lost, with MSH2/MSH6 deficient tumours having ∼46 mutations per megabase (mut/Mb) whilst MLH1/PMS2 deficient tumours had ∼25 mut/Mb [13]. Therefore, this further contributes to the evidence that the MMRd/MSI-h cohort is a heterogeneous population.

Clinically, MMRd patients are grouped solely based on the loss of one or more of the four MMR genes and there is no further stratification based on which protein is lost. Therefore, this study aimed to investigate if loss of the individual MMR genes differentially impacted factors associated with ICI response, namely PD-L1 expression, TMB and MSI. This work highlights that loss of the different MMR genes may contribute to heterogenous response rates to ICIs in the MMRd/MSI-h cohort.

## Materials & methods

### Cell lines

Cell lines were purchased from ATCC and grown at 37°C and 5 % CO_2_ in a humidified atmosphere. U2OS, OVCAR4, and SW620 were grown in Dulbecco's Modified Eagle Medium (DMEM; Gibco) whilst CT26 and SNU119 were grown in Roswell Park Memorial Institute 1640 (RPMI; Gibco) media. All media was supplemented with 10% fetal bovine serum (FBS; Invitrogen) and 100 U/ml penicillin and 100 μg/ml streptomycin (Gibco). The compounds cisplatin (Teva UK), IFNs (human IFNγ, Biolegend; murine IFNγ, Peprotech; IFNα, Abcam; IFNβ, Peprotech) and palbociclib (Selleck) were obtained commercially. To generate CRISPR-Cas9 guide control (gCTRL), MLH1 and MSH6 knock-out cell lines in the CT26 and U2OS, cells expressing Cas9-GFP were generated via lentiviral transduction and selected for with flow cytometry. Subsequently, the Dharmacon Edit-R system was used with predesigned CRISPR RNAs targeting MLH1 and MSH6. Single-cell clones were expanded, and screening was performed via western blot and targeted sequencing of the gRNA site. CT26 gCTRL, MLH1 KO and MSH6 KO cells were continuously passaged over 12 weeks, cryopreserving at weeks 1, 4, 8 and 12 in FBS + 10% DMSO. Cell populations from each time point could then be thawed simultaneously to allow for characterization and in MSH2 KO cells this has been shown to generate matched cell populations with MSI ranging from low to high. Unless otherwise stated, MMR KO cells were passaged for 12 weeks before characterisation to allow for the accumulation of mutations.

All cell lines were authenticated based on STR profile, viability, and morphologic inspection and were routinely mycoplasma tested.

### Protein analysis

Cell pellets were lysed in radioimmunoprecipitation assay (RIPA) buffer (50 nM Tris (pH 8), 150 nM NaCl, 1% NP40, 0.5% SDS) supplemented with protease inhibitors (Roche). For western blotting, lysates were electrophoresed on NuPAGE precast gels (Invitrogen) and immunoblotted with antibodies detailed in supplementary table 1. This was followed by incubation with anti-IgG-horseradish peroxidase and chemiluminescent detection (Supersignal West Pico Chemiluminescent Substrate, Pierce). Immunoblotting for β actin, β tubulin or vinculin (Cell signalling) was performed as a loading control. Protein densitometry was analysed using ImageJ and normalised to the loading control and control sample.

### Cell surface protein expression analysis

Cells were stained in suspension with an allophycocyanin-associated antibody targeting PD-L1 alongside an IgG2b, κ isotype control (Biolegend) on ice for 30 minutes. DAPI staining was also performed to exclude dead and apoptotic cells. Flow cytometry was performed using an LSR Fortessa 3-laser cell analyser (BD Biosciences). Unstained control samples were utilised to design the gating strategy applied to the experimental samples. Analysis was performed using FlowJo software (Tree Star) to quantify the relative cell surface expression by Median Fluorescence Intensity.

### qPCR

RNA was extracted from cells using the RNeasy kit (Qiagen). RNA was quantified using a nanodrop spectrophotometer (ThermoFisher) and 1 μg of RNA underwent reverse transcription using the High-Capacity cDNA Reverse Transcription kit (Applied Biosystems). Thermocycling was performed as follows: 25°C for 10 mins, 37°C for 120 mins then 85°C for 5 secs. 20 ng of RNA underwent qPCR using the TaqMan universal PCR master mix (Applied Biosystems) in the QuantStudio 5 system (Applied Biosystems). Samples were run in duplicate and thermocycling was carried out as follows: 50°C for 2 mins, 95°C for 10 mins then 40 cycles of 95°C for 15 secs and 60°C for 1 min. Analysis was performed using the 2 ^-ΔΔCT^ method normalised to β-actin or GAPDH.

### Cell cycle analysis

Cells were plated sparsely before treating with the CDK4/6 inhibitor palbociclib for 24 hours, which arrests the cells in G1. Cells were then collected and washed twice in PBS prior to fixing in 70% ethanol. Fixed cells were treated with RNase A to a final concentration of 50 μg/ml shaking at 37 °C for 30 minutes. The samples were then stained with propium iodide to a final concentration of 20 μg/ml for 30 minutes in the dark on ice before analysis with flow cytometry.

### Chromatin immunoprecipitation

Cells were crosslinked for 10 minutes at room temperature (RT) using formaldehyde (1% in 50 mM HEPES, 100 mM NaCl, 1 mM EDTA, 0.5 mM EGTA) before quenching with glycine (1.25 M) for 5 minutes at RT. Crosslinked cells were collected before being successively resuspended and rotated in 2 different lysis buffers (50mM Hepes–KOH pH 7.5, 140 mM NaCl, 1 mM EDTA, 10% Glycerol, 0.5% NP-40, 0.25% Triton X-100 and 10 mM Tris–HCL, pH 8, 200 mM NaCl, 1 mM EDTA, 0.5 mM EGTA). Sonication was performed in 10mM Tris–HCl pH 8, 100 mM NaCl, 1 mM EDTA, 0.5 mM EGTA, 0.1% Na–Deoxycholate and 0.5% N-lauroylsarcosine for 20 minutes with a duty factor of 5% (Covaris S220). All buffers contained protease inhibitors (Roche). Chromatin shearing analysis was performed following treatment with RNase and Proteinase K (Thermo) in TE buffer via electrophoresis. Chromatin was cleaned up by centrifuging with Triton X-100 (10%) before being quantified using DS DNA broad range kit (QUBIT). Dynabeads Protein A were washed and blocked in ice-cold BSA-PBS (0.5%) before incubation with MSH6 antibody (ThermoFisher) alongside an IgG control overnight at 4°C. Antibody-bound beads were washed in BSA-PBS (0.5%) before the addition of pre-cleared lysate in 1% Triton with protease inhibitor (Roche). Following rotation overnight at 4°C the beads were washed in RIPA (50 mM HEPES pH 7.6, 1 mM EDTA, 0.7% Na deoxycholate, 1% NP-40, 0.5 M LiCl) and TE buffer. The beads were then resuspended in elution buffer (50 mM Tris-HCL pH 8, 10 mM EDTA, 1% SDS) overnight at 65°C. RNase and proteinase K treatment was then performed before DNA was extracted using phenol-chloroform isoamyl alcohol and chloroform successively. Ethanol precipitation was used to purify DNA, and the pellet was resuspended in low EDTA TE buffer (10 mM Tris-HCl pH 8, 0.1 mM EDTA). 2 µl of immunoprecipitated DNA fragments in triplicate were quantified using SYBR green PCR master mix (Thermofisher) on the QuantStudio7 (Applied Biosystems). Primers designed by [14] targeting upstream of the PD-L1 gene start codon were used the sequences of which were as follows: − 1178 bp to − 1117 bp (forward 5′- GCT GGG CCC AAA CCC TAT T and reverse 5′-TTT GGC AGG AGC ATG GAG TT), − 455 bp to − 356 bp (forward 5′-ATG GGT CTG CTG CTG ACT TT and reverse 5′-GGC GTC CCC CTT TCT GAT AA-) and − 105 bp to − 32 bp (forward 5′-ACT GAA AGC TTC CGC CGA TT and reverse 5′-CCC AAG GCA GCA AAT CCA GT); hereafter referred to as primer 1, 2 and 3 respectively. Amplicons were between 60 and 150 base pairs. Results were normalised to the IgG using the 2 ^-ΔΔCT^ method.

### Whole exome sequencing

DNA was extracted from cells using the DNeasy blood and tissue kit (Qiagen) before whole exome sequencing (WES) was performed at Oxford Genomics (Wellcome) using Twist Library Preparation, yielding on average 115 million paired-end reads per sample with 150bp read length. After initial quality check using fastqc v0.11.5, reads were quality trimmed using trimgalore v0.6.5 and aligned to the mouse reference genome GRCm38 (mm10) using bwa v0.7.17. Picard v2.25.7 was used to mark read duplicates and assess insert size distributions. Base quality score recalibration was performed on known sites of variation using gatk v4.2.1.0 with mgp.v5.indels.pass.chr.sort.vcf.gz and 0-All.vcf.gz, downloaded from the Mouse Genome Project. Variant calling was performed using mutect2 [15]. Treated samples were compared to their respective controls and variant call format files were filtered using gatk before further annotating with annovar. Further filtering was applied for mutations with sequencing depth DP > = 10. MSI analysis was performed using MSIsensor pro v1.2.0, which records homopolymers of at least 5 bp length and microsatellites of maximum repeat unit length 5 from the reference genome [16,17]. The number given is the percentage of microsatellite sites with a somatic indel and an MSIsensor score of 3.5 is considered MSI. Normal WES data from a BALB/c spleen was downloaded from the Sequence Read Archive (National Centre for Biotechnology Information; sample SRR7774027) and processed as described above. The bam file was used as “normal” for the MSIsensor analysis. Raw sequencing data have been deposited to the NCBI SRA database under PRJNA1186681.

### Immunofluorescence

Cells were seeded on poly-L-lysine pre-coated coverslips (Corning). Following treatment, cells were incubated for 1 minute in 0.1% Triton (Sigma) in 1X PBS. Coverslips were then fixed with 4% paraformaldehyde (PFA; Booster) with 2% sucrose (Sigma) in 1X PBS for 20 minutes. This was followed by three washes in 1X PBS. Incubation with γH2AX antibody (Millipore) was performed in 2% BSA at 37°C for 45 minutes and washed 3 times with 1X PBS. Coverslips were then incubated with mouse secondary antibody (Invitrogen) in 2% BSA at 37°C for 30 minutes or at RT for 1 hour in the dark and washed 3 times with 1X PBS. Coverslips were then stained with DAPI for 1 minute, washed twice with 1X PBS and mounted onto slides with ProLong Gold antifade reagent (Invitrogen). Images were captured with Zeiss 710 confocal microscope and processed with ImageJ.

### Cell viability

Cells were seeded in a 96 well plate prior to indicated treatment. Following this, Cell Titre Glow (Promega) reagent was diluted 1:4 in PBS before removal of the growth medium and addition to the cells. The plate was shaken for 2 minutes before being allowed to incubate for 10 minutes in the dark at RT. Cell viability was then quantified by luminescence on a FLUORstar Omega plate reader (BMG Labtech).

### Proliferation assay

Cells were plated at a low density and placed in the Incucyte Live Cell Analysis system (Sartorius). The confluency mask was then applied to measure cell density over time, and this has been expressed as percentage confluency at each time point.

### Statistical analysis

Data represents standard error of the mean of at least three independent experiments. In most cases, 2-way ANOVA with a follow-up Tukey or Sidak test was used to determine statistical significance but where applicable 1-way ANOVA or unpaired student’s t-test was used as indicated in figure legends. The only exception to this is the MSIsensor data where a Fisher’s exact test was used with a follow-up Benjamini, Krieger and Yekutieli test to control for multiple testing (false discovery approach). Statistical analyses were performed using GraphPad Prism Software with p<0.05 regarded as significant.

## Results

### MSH6 loss does not lead to increased PD-L1 expression

**Fig. 1:**
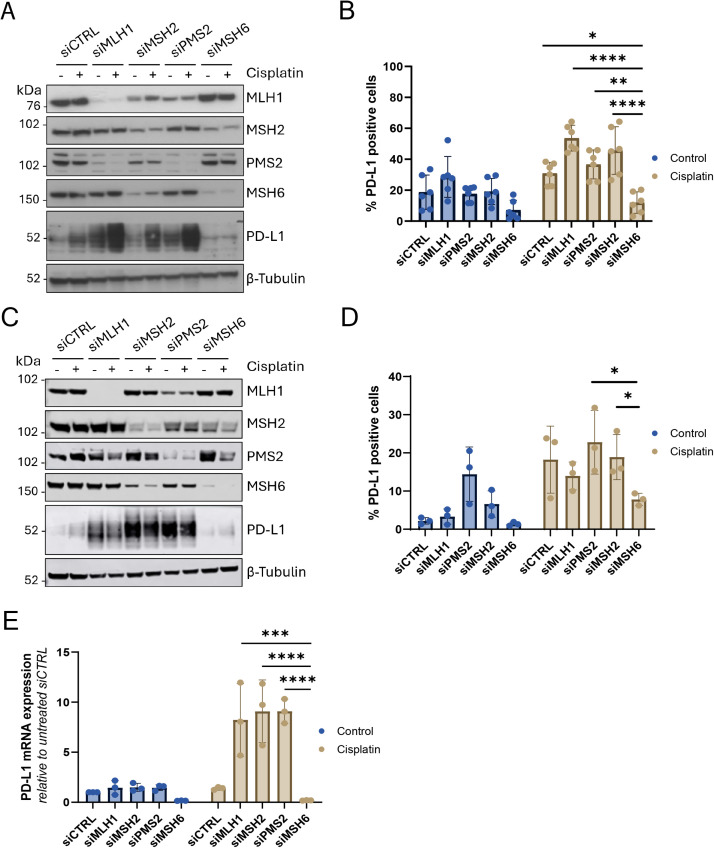
Depletion of MMR genes MLH1, MSH2 and PMS2, but not MSH6, results in increased expression of PD-L1. (A,B) OVCAR4 cells or (C,D) U2OS were transfected with siRNAs targeting each of the four MMR genes (MLH1, MSH2, PMS2 and MSH6) alongside a non-targeting siCTRL for 24 hours. The cells were then treated with cisplatin (2 μM) for 72 hours. (A,C) Western blot analysis to confirm knockdown of each MMR gene and measure PD-L1 expression. b tubulin was probed as a loading control. (B,D) Flow cytometry to measure PD-L1 expression. Analysis was performed using FlowJo with gating set up using an isotype control to determine the percentage of PD-L1 positive cells. Error bars represent (B) six or (D) three individual experiments. RNA was extracted and reverse transcribed into cDNA. Relative mRNA expression of PD-L1 was measured using RT-qPCR and normalized to the housekeeping gene b actin. The results were then normalized to the control sample using the 2 ^-ΔΔCT^ method. Error bars represent three independent experiments. (B,D,E) Statistical significance was carried out using two-way ANOVA with Tukey’s multiple comparison test (**** p<0.0001, ***p<0.001, ** p<0.01, * p<0.05).

Given the clinical responses to ICIs are variable among MMRd patients and PD-L1 expression has previously been used as a marker of ICI response, we initially investigated whether loss of different MMR genes could result in differential PD-L1 expression. To this end, human ovarian cancer cells (OVCAR4) and human osteosarcoma cells (U2OS) were transfected with small interfering RNA (siRNA) targeting MLH1, PMS2, MSH2 and MSH6 to transiently deplete expression. As a positive control, the cells were treated with cisplatin to induce DNA damage as this induces PD-L1 expression [18]. Induction of DNA damage was validated by performing γH2AX staining (Figure S1A,C). In addition, cell viability was assessed upon cisplatin treatment, to ensure that the majority of cells were still metabolically active despite DNA damage (Figure S1B,D). We observed that in cells depleted for MLH1, MSH2 or PMS2 there was increased expression of PD-L1 protein at both the basal level and following treatment with cisplatin (Fig. 1A,C). However, PD-L1 expression was not induced in the MSH6-depleted cells. PD-L1 expression was also investigated at the cell surface level as this is where PD-L1 exerts its function. At the cell surface level, PD-L1 expression was significantly increased in the MLH1, MSH2 and PMS2 silenced cells in comparison to the MSH6 silenced cells following treatment with cisplatin in both OVCAR4 (Fig. 1B; siMLH1, siMSH2 vs siMSH6 p<0.0001, siPMS2 vs siMSH6 p<0.01) and U2OS (Fig. 1D; U2OS siPMS2, siMSH2 vs siMSH6 p<0.05) cell lines. This was also validated at the mRNA level, and it was observed that following treatment with cisplatin PD-L1 mRNA expression was significantly higher in MLH1, MSH2 and PMS2 silenced cells in comparison to the MSH6 silenced cells (Fig. 1E; siMLH1 vs siMSH6 p<0.001; siMSH2, siPMS2 vs siMSH6 p<0.0001).

It is worth noting that it has previously been shown that upon loss of MSH2, the expression of MSH6 is reduced, although the mechanism for this is unclear [19,20]. We observed reduced MSH6 expression in the siMSH2 transfected cells (Figure S2A-D), but unlike the siMSH6 transfected cells, we observed an increase in PD-L1 expression upon MSH2 loss. This suggests that this phenotype is restricted to isolated MSH6 loss rather than the reduction in MSH6 expression as a result of MSH2 loss.

Given the clinical responses to ICIs are variable among MMRd patients and PD-L1 expression has previously been used as a marker of ICI response, we initially investigated whether loss of different MMR genes could result in differential PD-L1 expression. To this end, human ovarian cancer cells (OVCAR4) and human osteosarcoma cells (U2OS) were transfected with small interfering RNA (siRNA) targeting MLH1, PMS2, MSH2 and MSH6 to transiently deplete expression. As a positive control, the cells were treated with cisplatin to induce DNA damage as this induces PD-L1 expression [18]. Induction of DNA damage was validated by performing γH2AX staining (Figure S1A,C). In addition, cell viability was assessed upon cisplatin treatment, to ensure that the majority of cells were still metabolically active despite DNA damage (Figure S1B,D). We observed that in cells depleted for MLH1, MSH2 or PMS2 there was increased expression of PD-L1 protein at both the basal level and following treatment with cisplatin (Fig. 1A,C). However, PD-L1 expression was not induced in the MSH6-depleted cells. PD-L1 expression was also investigated at the cell surface level as this is where PD-L1 exerts its function. At the cell surface level, PD-L1 expression was significantly increased in the MLH1, MSH2 and PMS2 silenced cells in comparison to the MSH6 silenced cells following treatment with cisplatin in both OVCAR4 (Fig. 1B; siMLH1, siMSH2 vs siMSH6 p<0.0001, siPMS2 vs siMSH6 p<0.01) and U2OS (Fig. 1D; U2OS siPMS2, siMSH2 vs siMSH6 p<0.05) cell lines. It is worth noting that there is a loss of co-expression observed between MSH2 and MSH6 meaning that in MSH2 silenced cells there is also a reduction in MSH6 expression and in MSH6 silenced cells there is also a reduction in MSH2 expression (Figure S2). The phenotype we observe of failure to induce PD-L1 expression is restricted to isolated loss of MSH6 expression.

**Fig. 2:**
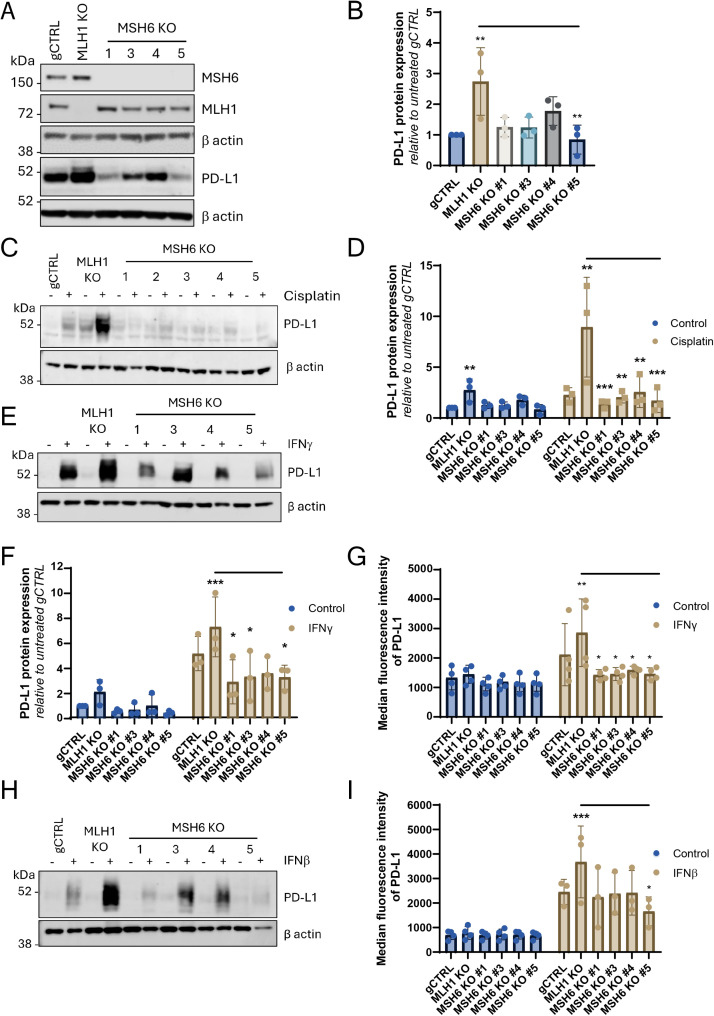
MSH6 is required for induction of PD-L1 expression following treatment with cisplatin and interferons. Cells were treated with (C,D) cisplatin (2 mM), (E-G) IFNg (50 U/ml) or (H,I) IFNb (100 u/ml) for 72 hours. Western blot analysis to (A) confirm knockout of MSH6 and MLH1 and (A,C,E) probe for PD-L1 expression. b actin was probed as a loading control. N=3. A representative blot is shown. (D,F) Protein densitometry was analysed using ImageJ. The intensity of PD-L1 expression was measured and normalised to β-actin. The normalised expression was then normalised to the untreated control cells. Error bars represent three individual experiments. (G,I) Flow cytometry to measure PD-L1 expression. Analysis was performed using FlowJo with gating set up using an isotype control to determine the median fluorescent intensity of PD-L1 positive cells. Error bars represent (G) four or (I) three individual experiments. (B,F,G,I) Statistical significance was determined using 2-way ANOVA with Tukey’s multiple comparison test and is in comparison to gCTRL unless otherwise shown (***p<0.001, **p<0.01, *p<0.05, no * = not significant).

To corroborate these findings and ensure this difference in PD-L1 expression was not an effect of siRNA transfection, CRISPR-Cas9 was utilised to knockout (KO) MLH1 and MSH6 in the murine CRC cell line CT26 (Figure S3) and in U2OS cells (Fig. 2A). We observed that MLH1 KO increased basal PD-L1 expression relative to both the gCTRL and MSH6 KO cells (Fig. 2A,B; p<0.01), therefore, consistent with siRNA knockdown, MSH6 KO failed to increase PD-L1 expression in U2OS cells. This was conserved in the murine CT26 cells where there was also higher PD-L1 expression in the MLH1 KO cells in comparison to the gCTRL and MSH6 KO cells (Figure S3; p<0.05). Due to the loss of co-expression that has previously been observed between MSH2 and MSH6 [20], MSH2 expression in the MSH6 KO cells was investigated and it was observed that, although MSH2 expression was reduced, it was still expressed (Figure S2E,F).

### PD-L1 expression is not induced in MSH6-deficient cells upon DNA damage or IFN treatment

The induction of DNA damage is a known regulator of PD-L1 expression [18]. Therefore, we next treated MLH1 and MSH6 KO cells with the alkylating agent cisplatin, to induce DNA damage and, observed that whilst there was a significant induction of PD-L1 protein expression in the MLH1 KO U2OS cells (Fig. 2C,D; p<0.01), this induction was not observed in the MSH6 KO cells. In addition, in the cisplatin-treated samples, PD-L1 expression was significantly reduced in the MSH6 KO cells when compared to the MLH1 KO cells (Fig. 2C,D; MSH6 KO #1 and #5 p<0.001, MSH6 KO #3 and #4 p<0.01). Similarly, CT26 MSH6 KO cells failed to induce PD-L1 expression to the same extent as gCTRL or MLH1 KO cells following treatment with cisplatin (Figure S4A,B). Therefore, consistent with what was observed upon siRNA transfection there was a lack of induction upon PD-L1 expression in MSH6-deficient cells, which was conserved between mouse and human. PD-L1 expression was also validated at the mRNA level, and it was observed that following treatment with cisplatin, PD-L1 expression was induced significantly more in MLH1 KO cells in comparison to MSH6 KO cells (Figure S4C; MLH1 KO vs MSH6 KO #1,3-5 p<0.0001).

Next, we wanted to investigate whether these differences in PD-L1 expression were specific to DNA damage. To this end, treatment with IFNs was utilised as an alternative method to induce PD-L1 expression [21]. We observed that IFNγ significantly induced PD-L1 expression in the MLH1 KO cells at both the protein and cell surface level (Fig. 2E-G; protein p<0.001, cell surface p<0.01). However, upon treatment with IFNγ in MSH6 KO cells, there was a failure to induce PD-L1 expression to the same extent resulting in significantly less PD-L1 protein expression in comparison to the MLH1 KO cells (Fig. 2F,G; protein MSH6 KO #1,3,5 p<0.05, cell surface p<0.05). Similarly, CT26 MSH6 KO cells failed to significantly induce PD-L1 expression whilst significant PD-L1 induction was observed in gCTRL and MLH1 KO cells following treatment with IFNγ (Figure S4E,F; gCTRL p<0.05, MLH1 KO p<0.01). PD-L1 expression was also validated at the mRNA level and it was observed that following treatment with IFNγ, PD-L1 expression was induced significantly more in MLH1 KO cells in comparison to MSH6 KO cells (Figure S4D; MLH1 KO vs MSH6 KO #5 p<0.05).

In cells silenced for MLH1, increased PD-L1 induction was observed following both IFNα and IFNβ treatment in comparison to the MSH6 KO cells (Figure S4G,H). This was further validated in the MLH1 KO cells where we observed that PD-L1 protein and cell surface expression was more strongly induced than in the gCTRL and MSH6 KO cells (Fig. 2H,I; p<0.001). Furthermore, the IFNβ-treated MSH6 KO cells had significantly lower PD-L1 expression at the cell surface in comparison to the MLH1 KO cells (MSH6 KO #5 p<0.05). PD-L1 expression was also validated at the mRNA level, and it was observed that following treatment with cisplatin, PD-L1 expression was induced significantly more in MLH1 KO cells in comparison to MSH6 KO cells (Figure S4G; MLH1 KO vs MSH6 KO #1,3,5 p<0.0001; MLH1 KO vs MSH6 KO #4, p<0.001). PD-L1 expression was also validated at the mRNA level, and it was observed that following treatment with cisplatin, PD-L1 expression was induced significantly more in MLH1 KO cells in comparison to MSH6 KO cells (Figure S4I; MLH1 KO vs MSH6 KO #1,3,5 p<0.0001; MLH1 KO vs MSH6 KO #4, p<0.001).

Taken together, our data suggests that failure to induce PD-L1 expression to the same extent upon MSH6 loss in comparison to upon loss of other MMR proteins is not related to DNA damage but may define a non-canonical role for MSH6 in the regulation of PD-L1 expression.

### Differential PD-L1 expression does not correlate with TMB or JAK/STAT signalling

**Fig. 3:**
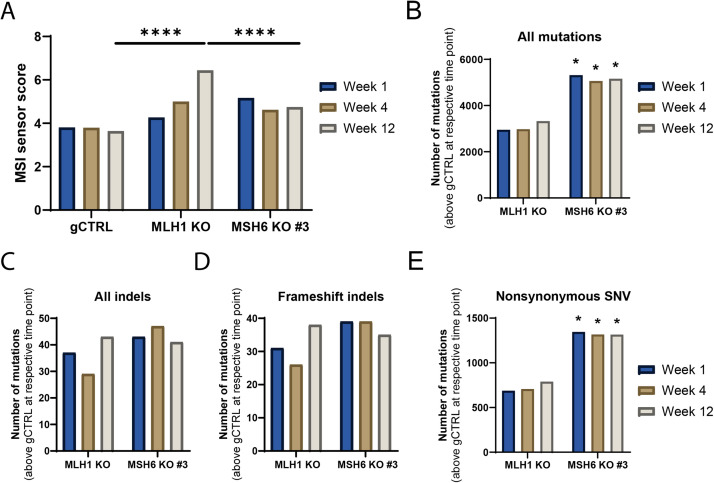
PD-L1 expression, MSI and TMB do not correlate in MSH6 KO cells. DNA was extracted and sent to Oxford Genomics for whole exome sequencing before analysis was carried out to determine (A) MSI using MSIsensor and (B-E) TMB using Mutect2. (A) Statistical analysis was determined using Fisher’s exact test with a follow-up Benjamini, Krieger and Yekutieli test to correct for multiple testing (****p<0.0005). (B-E) Statistical analysis was determined using 2-way ANOVA with Tukey’s multiple comparison test (MSH6 KO vs. MLH1 KO; *p<0.05, no * = not significant).

To determine whether PD-L1 expression correlated with MSI and TMB, whole exome sequencing was carried out on CT26 MSH6 KO and MLH1 KO cells, which were serially passaged over 12 weeks in parallel with gCTRL cells to allow the accumulation of mutations [6]. A proliferation assay was also performed to ensure that the gCTRL, MLH1 KO and MSH6 KO cells proliferated at a similar rate as differential proliferation rates could account for any differences in accumulated mutations that are observed (Figure S6). A proliferation assay was also performed to ensure that the gCTRL, MLH1 KO and MSH6 KO cells proliferated at a similar rate as differential proliferation rates could potentially account for any differences in accumulated mutations that are observed (Figure S6). In MLH1 KO matched cell populations from weeks 1, 4 and 12, we observed that MSI increased over time so that at week 12 there was significantly more MSI in comparison to gCTRL cells (Fig. 3A, p<0.0001). This is consistent with what has been previously observed in MSH2 KO cells [6]. However, in the MSH6 KO cells, there was no increase in MSI over time, such that at week 12, MSI was significantly higher in the MLH1 KO in comparison to the MSH6 KO cells (Fig. 3A, p<0.0001). Interestingly, this suggests that MSI may correlate with PD-L1 expression. To investigate whether MSI correlated with TMB, the total number of mutations compared to the gCTRL was quantified. Surprisingly, we observed that MSH6 KO cells had significantly higher TMB than MLH1 KO cells (Fig. 3B, p<0.05), and this did not increase over time. To understand this further we analysed the different types of mutations generated and observed that no significant differences were observed between all indels or frameshift indels when comparing the MLH1 KO and MSH6 KO cells (Fig. 3C,D). However, MSH6 KO cells had significantly more nonsynonymous single nucleotide variants (SNVs) than the MLH1 KO cells (p<0.05, Fig. 3E).

**Fig. 4:**
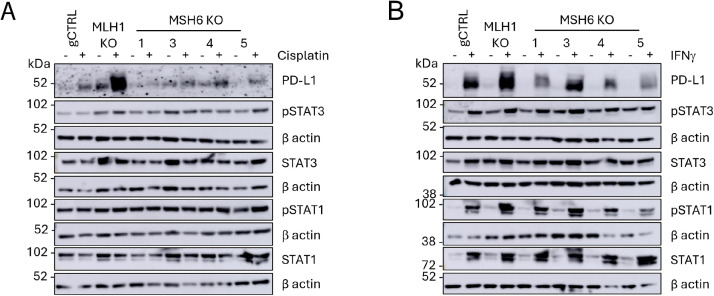
JAK-STAT signalling is not differentially regulated upon loss of MLH1 and MSH6. U2OS gCTRL, MLH1 KO and MSH6 KO were treated with (A) cisplatin (2 μM) or (B) IFNg (50 U/ml) for 72 hours. Western blot analysis was performed to probe for PD-L1 as well as STAT1/3 and their phosphorylated form (S727), which is required for the full transcriptional activity and biological function of STAT1/3. b actin was probed as a loading control. N=3. A representative blot is shown.

JAK/STAT signalling is central to PD-L1 regulation in response to DNA damage and IFNs [[18], [21]]. Therefore, to determine whether these differences in PD-L1 expression upon MLH1 and MSH6 loss were mediated by JAK/STAT signalling the expression and phosphorylation of STAT1 and STAT3 were investigated. The U2OS MLH1 KO and MSH6 KO cells were treated with cisplatin (Fig. 4A) and IFNγ (Fig. 4B) to activate STAT1/3 phosphorylation. We observed that there were no differences in the expression or phosphorylation of STAT1/3 upon MSH6 loss. Despite this, decreased expression of STAT1 as well as expression and phosphorylation of STAT3 was observed in the CT26 MSH6 KO cells in comparison to the MLH1 KO cells (Figure S5). However, as this was not observed in the U2OS cells we concluded that this was not the conserved mechanism mediating the reduced PD-L1 expression observed in MSH6 KO cells. As these differences were not observed to be specific to DNA damage and instead indicated an inability to induce PD-L1 expression in MSH6 deficient cells, the possibility that MSH6 could directly regulate PD-L1 expression was investigated.

### MSH6 can bind directly to the PD-L1 promoter

**Fig. 5:**
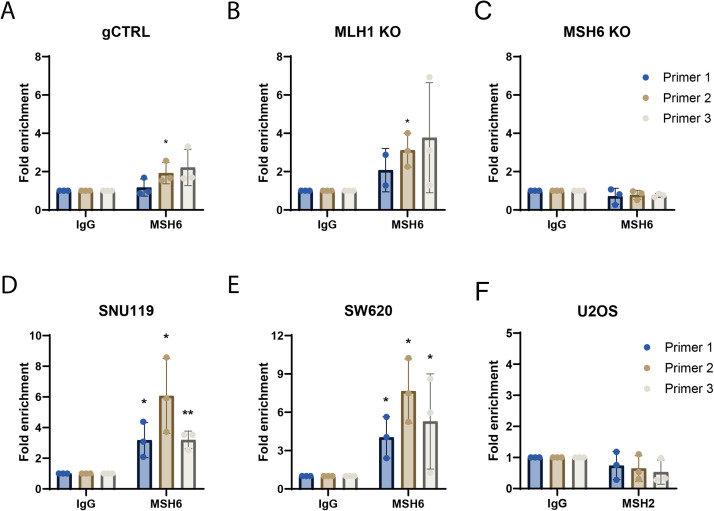
MSH6 binds directly to the PD-L1 promoter. Chromatin immunoprecipitation was performed using anti-IgG and anti-MSH6 conjugated beads on crosslinked and sonicated chromatin from U2OS (A) gCTRL, (B) MLH1 KO (C) MSH6 KO and (F) wildtype cells as well as (D) SW620 and (E) SNU119 cells. Primers targeting the PD-L1 promoter were then used in qPCR to quantify pulled-down DNA. Results are represented as fold change compared to the IgG. Error bars represent three individual experiments. Statistical analysis was determined using an unpaired t-test (**p<0.01, *p<0.05, no * = not significant).

To determine whether MSH6 was binding directly to the PD-L1 promoter in the U2OS gCTRL, MLH1 KO and MSH6 KO cells, we performed chromatin immunoprecipitation (ChIP) by immunoprecipitating MSH6 coupled with qPCR utilising primers spanning the PD-L1 promoter. Our analysis revealed that there was significant binding of MSH6 to the PD-L1 promoter in gCTRL cells (Fig. 5A; primer 2 p<0.05). Interestingly, in MLH1 KO cells statistically significant binding of MSH6 to the PD-L1 promoter was also observed (Fig. 5B; p<0.05). This was also performed in MSH6 KO cells to act as a negative control and no binding was observed (Fig. 5C).

The binding of MSH6 to the PD-L1 promoter was further validated in two additional human cancer cell lines, including SW620 (colorectal cancer) and SNU119 (ovarian cancer). In SNU119 cells, we observed significant binding of MSH6 to the PD-L1 promoter in all three primer regions (Fig. 5D, primer 1,2 p<0.05; primer 3 p<0.01). This was also true in the SW620 cells (Fig. 5E; p<0. 05). Therefore, our results strongly suggest that the binding of MSH6 to the PD-L1 promoter is a conserved phenotype.

To determine whether MSH6 binds to PD-L1 as part of the MutSα heterodimer, we carried out ChIP analysis by immunoprecipitating MSH2 followed by qPCR utilising primers spanning the PD-L1 promoter. However, we did not observe MSH2 binding to the PD-L1 promoter (Fig. 5F). This suggests that MSH6 is not bound as part of the MutSα (MSH2-MSH6) complex.

### MSH6 can bind to the PD-L1 promoter in a SETD2-dependent manner

**Fig. 6:**
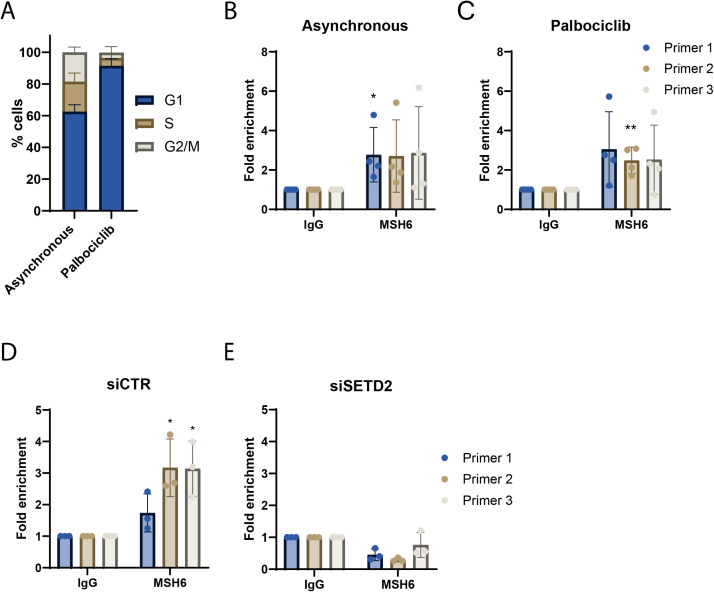
MSH6 binds to the PD-L1 promoter in a SETD2-dependent manner in G1. (A-C) Cells were arrested in G1 using the CDK4/6 kinase inhibitor palbociclib and (A) cell cycle analysis was performed using propidium iodide staining, which was quantified using flow cytometry to confirm arrest. (D,E) U2OS cells were transfected with siRNA targeting SETD2 alongside a non-targeting control (siCTRL). (B-E) Chromatin immunoprecipitation was performed using anti-IgG and anti-MSH6 conjugated beads on crosslinked and sonicated chromatin from U2OS wild-type cells. Primers targeting the PD-L1 promoter were then used in qPCR to quantify pulled-down DNA. Results are represented as fold change compared to the IgG. Error bars represent (A,D,E) three or (B,C) four individual experiments. Statistical analysis was determined using an unpaired t-test (**p<0.01, *p<0.05, no * = not significant).

To further understand the role of MSH6 in the regulation of PD-L1 expression, we next investigated which phase of the cell cycle MSH6 was bound to the PD-L1 promoter. The canonical role of MSH6 in MMR occurs during S phase to repair DNA replication errors [22]. Therefore, the binding of MSH6 to the PD-L1 promoter in G1 would suggest a role in transcription instead of MMR. To this end, U2OS cells were arrested in G1 phase using the cyclin-dependent kinase (CDK)4/6 inhibitor, palbociclib (Fig. 6A) followed by ChIP analysis of MSH6 at the PD-L1 promoter. We observed significant binding of MSH6 in both the WT asynchronous cells (Fig. 6B; primer 1 p<0.05) and the G1-arrested cells (23C; primer 2 p<0.01).

Thus far, we have demonstrated that MSH6 is required for induction of PD-L1 expression and that it binds to the PD-L1 promoter during G1, suggesting a role in transcription. However, given no previous role for MSH6 in transcription has been defined, the mechanism of recruitment is unclear. MutSα can be recruited to chromatin via the trimethyl histone mark on lysine 36 of histone 3 (H3K36me3), which is made by the histone trimethylase SETD2 (X. )[24]. This histone mark can be bound by the Proline-Tryptophan-Tryptophan-Proline (PWWP) domain in MSH6. We hypothesised that this could also be the mechanism of recruitment for MSH6 to the PD-L1 promoter. To investigate this, we silenced SETD2 in U2OS cells before performing ChIP analysis to determine if MSH6 binding to the PD-L1 promoter was dependent on SETD2. We observed that upon siRNA-mediated silencing of SETD2, there was no binding of MSH6 to the PD-L1 promoter (Fig. 6E) whilst in the siCTRL transfected cells significant binding was observed (Fig. 6D; primer 2,3 p<0.01). Therefore, this suggests that the histone mark H3K36me3, which is made by SETD2 and can be bound by the PWWP domain at the N terminus of MSH6, is involved in the recruitment of MSH6 to the PD-L1 promoter. We hypothesised that if SETD2 is required for recruiting MSH6 to the PD-L1 promoter then SETD2 loss may result in a similar phenotype to MSH6 loss, such that there would be a reduction in PD-L1 induction. To this end, gCTRL, MLH1 KO and MSH6 KO cells were transfected with siRNA targeting SETD2 and treated with IFNγ but reduced PD-L1 expression upon silencing of SETD2 was not observed (Figure S7). This is likely due to the fact that, as the sole histone methyltransferase for histone H3.1 and H3 variants, SETD2 has been linked to organising chromatin structure, alternative splicing and the DNA damage response [25]. Therefore, the impact of SETD2 loss on these other pathways may be influencing the expression of PD-L1 as well.

Overall, our data suggests that MSH6 deficient cells fail to induce PD-L1 expression at the basal level and to the same extent as upon loss of other MMR proteins in response to DNA damage and IFN treatment. We have shown that this is due to the ability of MSH6 to bind directly to the PD-L1 promoter in a SETD2-dependent manner to regulate PD-L1 transcription.

## Discussion

Since the approval of MMRd/MSI-h as a biomarker for response to pembrolizumab, the potential for ICIs to effectively treat MMRd/MSI-h tumours has been demonstrated in many studies ([26]; Aurelien [[27], [28], [29]]). However, ∼40-70% of MMRd/MSI-h patients remain refractory to ICI treatment, highlighting the inadequacy of this biomarker alone in predicting patients likely to respond to ICIs [5]. This variability in response is not only true for the MMRd/MSI-h cohort but also for the other approved biomarkers for predicting response to ICIs, namely TMB and PD-L1 expression [[30], [31], [32]]. It’s also important to consider that these three approved biomarkers for predicting response to ICIs, although biologically interlinked, do not necessarily correlate in patients [7]. There is context dependency to this, for example, PD-L1 expression and high TMB are significantly associated in gastric and endometrial cancers, which may explain why ICIs are much more successful in some cancer types than others [10]. Therefore, it is evident that more work is required to understand the biological mechanisms promoting response to ICIs to allow for better patient stratification in the clinic. Hence in this study, MSI, TMB and PD-L1 expression were evaluated in MMRd cells to identify heterogeneity and elucidate the mechanism behind this.

Pembrolizumab, the approved ICI to treat MMRd/MSI-h patients, targets the PD-1/PD-L1 interaction, and PD-L1 expression on tumour cells has been approved as a biomarker for predicting response to ICIs in some cancer types, such as NSCLC [33]. This was based on the KEYNOTE-024 phase III trial, which found that in advanced NSCLC, pembrolizumab as a first-line treatment in patients with >50% of PD-L1 positive tumour cells achieved an improved overall survival (5.2 months vs 4.1 months) and ORR (44.8% vs. 27.8%) in comparison to platinum-based chemotherapy [34]. However, another phase III trial observed prolonged survival in patients treated with anti-PD-1 instead of chemotherapy regardless of PD-L1 expression [35]. Therefore, although tumoral PD-L1 expression is not intrinsically linked with response to ICIs in that it is not sufficient for response, it has been associated with response to ICIs. It is also important to note that PD-L1 can be expressed on immune cells, and it has been shown that quantifying PD-L1 on all cells in the tumour microenvironment, rather than just on the tumour cells, can better predict response to pembrolizumab [36]. We have demonstrated that MSH6 deficient cells fail to induce PD-L1 expression to the same extent as MLH1, PMS2 and MSH2 deficient cells due to the ability of MSH6 to directly regulate PD-L1 expression. We also observed that MSH2 was not bound to the PD-L1 promoter, which suggests that MSH6 is not acting as part of MutSα. This may explain why we see reduced PD-L1 expression in MSH6 deficient cells but not in MSH2 deficient cells even though MSH2 and MSH6 heterodimerise to perform their function in MMR.

The phenotype we observe of failure to induce PD-L1 expression upon loss of MSH6 expression to the same extent as upon loss of MSH2, PMS2 and MLH1 expression is observed to be specific to isolated loss of MSH6. The reduced MSH6 expression upon loss of MSH2 does not result in the same phenotype. Interestingly, beyond response to ICIs there is accumulating evidence that loss of the different MMR genes can result in distinct phenotypes. Particularly relevant to this work, a recent study identified stochastic switching between activation and inactivation of MSH6 and MSH3 as a driver of intratumoural heterogeneity, with loss of MSH6/MSH3 resulting in a hypermutator phenotype that drives subclonal evolution at the cost of cell fitness [37]. This allows the acquisition of traits for immune evasion and clone expansion, followed by a reversion in the inactivation of MSH6/MSH3 that stabilises the mutation rate to restore cell fitness. This results in subclonal neoantigen complexity, which in other studies has been linked to a lack of ICI response [38]. Similar to what we have observed, the phenotype they describe in this study is restricted to isolated MSH6/MSH3 loss as opposed to MSH2 loss.

Further evidence that isolated loss of MSH6 results in a distinct phenotype can be derived from Lynch syndrome (LS), which is caused by germline mutations in any of the MMR genes. LS is an autosomal condition that increases a patient’s risk of colorectal and endometrial cancer to ∼80% and 40%, respectively [39]. Interestingly, it is a highly heterogeneous patient population with varying penetrance of cancer, age of onset and molecular profiles. This has been attributed, at least in part, to loss of the different MMR genes and, following recent data from the Prospective LS Database, gene-specific stratification of LS patients has been proposed [[40], [41], [42]]. For example, in contrast to MLH1- and MSH2-associated, MSH6-associated LS is sex limited and only has high penetrance in females with a high incidence of ovarian and endometrial cancer that occurs at an older age than in MLH1 and MSH2-associated LS. Alongside this, numerous studies that have identified heterogeneity in the mutational profiles upon loss of different MMR genes, for example, CTNNB1 mutations have been found to occur at a much lower rate in MSH6-mutated CRCs in comparison to MLH1-mutated CRCs [41]. It has also been demonstrated that MSH6-deficient CRCs have significantly higher MMRd signature-associated SNVs than PMS2-deficient CRCs [43]. This is consistent with our whole exome sequencing analysis, which demonstrated that CT26 MSH6 KO cells had a much higher burden of nonsynonymous SNVs in comparison to CT26 MLH1 KO cells. In contrast, it has previously been observed in several studies that MSH6 deficient tumours have lower MSI than other MMRd tumours or are microsatellite stable [[23], [44], [45], [46], [47]]. Similarly, we observed that in MSH6 KO cells, MSI did not increase over time and that in week 12 cells MLH1 KO cells had significantly higher MSI than MSH6 KO cells. Overall, there is compelling evidence that loss of the different MMR genes, including isolated MSH6 loss, can result in distinct phenotypes and that this warrants further study, particularly in relation to ICI response.

We have also shown that MSH6 recruitment to the PD-L1 promoter is dependent on SETD2, a histone trimethyltransferase that has roles in alternative splicing, chromatin architecture organisation and homologous recombination [[25], [48]]. Interestingly, SETD2 deficient cells have many characteristics of MMRd cells, including high TMB and MSI, and SETD2 dysfunction has been associated with improved response to ICIs independent of TMB [[49], [50], [51], [52]]. This phenotype is likely caused at least in part by an impairment of MMR due to an inability to recruit MutSα to the chromatin via the PWWP domain on MSH6 (F. )[50]. Here, we have shown that MSH6, but not MSH2, is observed binding to the PD-L1 promoter in a SETD2-dependent manner in G1, which points more towards a role in transcription. Therefore, further work is warranted to better define this novel role for MSH6 as a transcription factor and to determine whether MSH6 can influence the transcription of other proteins.

TMB has also received separate site agnostic approval for treatment with pembrolizumab following the KEYNOTE-158 trial, where high TMB tumours (>10 mut/Mb) achieved an ORR of 29% compared to 6% in the low TMB group (<10 mut/Mb) [11]. Yet with studies showing that ∼60-85% of high TMB patients do not achieve an ORR following treatment with ICIs [30], it is evident that although mutational load is associated with response to ICIs, it is not sufficient to elicit response. It has been suggested that instead of overall TMB being key, it’s more a matter of ‘quality over quantity’ with certain mutations, such as indels, evoking a stronger immune response than other types of DNA damage [53]. This is consistent with the data that has suggested that response to ICIs in MMRd patients correlates with the degree of MSI [6]. This is also in line with the better response rates observed in MMRd/MSI-h patients when compared to the more general TMB-h, as MSI results in indels and, therefore, more immunogenic mutations. Interestingly, even though we observed that MSH6 KO cells had a higher TMB in comparison to MLH1 KO cells, this was primarily comprised of SNVs, which has also been observed in the clinic [41]. The accumulation of SNVs in MSH6 deficient cells could be because MutSα is responsible for recognising SNVs whilst MutSβ is responsible for recognising larger indels [54]. Therefore, if it is ‘quality over quantity’ when it comes to mutations driving response to ICIs and the mutational profiles of MMRd/MSI-h patients are not homogenous, this could be driving variable response rates to ICIs in this patient cohort. Similarly, as mentioned previously, there is evidence that MSH6 deficient tumours have lower MSI or are actually microsatellite stable [[23], [44], [45], [46], [47]]. Therefore, if the degree of MSI is heterogeneous within the MMRd population then the classification of MMRd and MSI-h as a single patient cohort could be detrimental to identifying patients that are going to derive clinical benefit from ICIs.

Interestingly, beyond response to ICIs there is accumulating evidence that demonstrates that loss of the different MMR genes can result in distinct phenotypes. This includes the fact that noncanonical roles for MMR proteins have been identified, for example, MLH1 has been implicated in regulating mitochondrial metabolism with loss of MLH1 resulting in reduced basal oxygen consumption rate and reduced spare respiratory capacity [55]. In addition, particularly relevant to this work, a recent study identified stochastic switching between activation and inactivation of MSH6 and MSH3 as a driver of intratumoural heterogeneity, with loss of MSH6/MSH3 resulting in a hypermutator phenotype that drives subclonal evolution at the cost of cell fitness [37]. This allows the acquisition of traits that allow immune evasion and clone expansion, followed by a reversion in the inactivation of MSH6/MSH3 that stabilises the mutation rate to restore cell fitness. This results in subclonal neoantigen complexity, which in other studies has been linked to a lack of ICI response [38]. Similar to what we have observed, the phenotype they describe in this study is restricted to isolated MSH6/MSH3 loss as opposed to MSH2 loss. Therefore, this suggests that isolated MSH3/MSH6 loss could be a phenomenon that warrants further study, including in relation to ICI response.

In summary, the variability in response to ICIs that is observed in the MMRd/MSI-h population may be derived from the loss of the different MMR genes. Here, we have shown that MSH6 KO cells have a lower expression of PD-L1 and an inability to induce expression to the same extent as other MMRd cells. We propose that this is due to the ability of MSH6 to bind directly to the PD-L1 promoter in a SETD2-dependent manner, defining a novel role for MSH6 as a transcription factor. In addition, we have demonstrated that whilst MSI is lower in MSH6 deficient cells in comparison to MLH1 deficient cells, the reverse is true for TMB. This suggests that MSI but not TMB correlates with PD-L1 expression as MSH6 KO cells have a higher TMB but not MSI when compared to MLH1 KO cells. Therefore, this study contributes to the accumulating evidence that gene-specific stratification of MMRd patients could be therapeutically and clinically beneficial.

**Supplementary Figure 1. Cisplatin induces gH2AX formation in U2OS and OVCAR4 cells, but the majority of cells remain viable.** (A) U2OS and (C) OVCAR4 cells were seeded on poly-L-lysine coated coverslips prior to treatment with 2 mM cisplatin for 72 hours. Cells were fixed with 4% paraformaldehyde, stained with anti-gH2AX antibody and DAPI, and mounted on microscope slides. Images were acquired on LSM710 confocal microscope. N=3. Representative images are shown. (B) U2OS and (D) OVCAR4 cells were treated with 2 mM cisplatin for 72 hours prior to a Cell Titre Glow assay being performed. N=3.

**Supplementary Figure 2. Loss of co-expression between MSH2 and MSH6.** (A,B) OVCAR4 cells or (C,D) U2OS were transfected with siRNAs targeting each of the four MMR genes (MLH1, MSH2, PMS2 and MSH6) alongside a non-targeting siCTRL. Following western blot analysis, protein densitometry was analysed using ImageJ. The intensity of (A,C) MSH2 or (B,D) MSH6 expression was measured and normalised to β-actin. The normalised expression was then normalised to the siCTRL cells. Error bars represent three individual experiments. (E) CT26 gCTRL, MLH1 KO and MSH6 and (F) U2OS gCTRL and MSH6 KO cells were analysed by western blot to probe for MSH2 expression. b-actin was probed as a protein loading control. Three independent experiments were performed, and a representative blot is shown.

**Supplementary Figure 3. CT26 MLH1 KO cells have higher PD-L1 expression than MSH6 KO cells.** (A) Western blot analysis was performed on CT26 gCTRL, MLH1 KO and MSH6 KO cells to probe for MLH1, MSH6 and PD-L1. b actin was probed as a loading control. N=3. A representative blot is shown. (B) Protein densitometry was analysed using ImageJ. The intensity of PD-L1 expression was measured and normalised to β-actin. The normalised expression was then normalised to the untreated control cells. Error bars represent three individual experiments. Statistical analysis was determined using 1-way ANOVA (*p<0.05, no * = not significant).

**Supplementary figure 4. MSH6 deficient cells fail to induce PD-L1 expression to the same extent as MLH1 deficient cells.** (A,B,E,F) CT26 or (C,D,G-I) U2OS gCTRL, MLH1 KO and MSH6 KO cells were treated with (A-C) cisplatin (2 μM), (B,D-F) IFNg (50 U/ml) or (I) IFNa (500 U/ml) for 48 hours (CT26) or 72 hours (U2OS). (A,E) Western blot analysis was performed to blot for PD-L1. b actin was probed as a loading control. N=3. A representative blot is shown. (C,D,I) RNA was extracted and reverse transcribed into cDNA. Relative mRNA expression of PD-L1 was measured using RT-qPCR and normalized to the housekeeping gene b actin. The results were then normalized to the control sample using the 2 ^-ΔΔCT^ method. Error bars represent three independent experiments. Statistical significance was determined using 2-way ANOVA with Tukey’s multiple comparison test and is in comparison to gCTRL unless otherwise shown (****p<0.0001, ***p<0.001, *p<0.05, no * = not significant). (G,H) U2OS cells were transfected with siRNA targeting MLH1 and MSH6 alongside a non-targeting control (siCTRL) prior to treatment with (G) IFNa (500 U/ml) or (D) IFNb (100 U/ml) for 24 hours. Western blot analysis was performed to blot for PD-L1 as well as MSH6 and MLH1 to confirm knockdown. b actin was probed as a loading control. N=3. A representative blot is shown.

**Supplementary Figure 5. JAK-STAT signalling is not differentially regulated upon loss of MLH1 and MSH6.** CT26 gCTRL, MLH1 KO and MSH6 KO were treated with (A) cisplatin (2 μM) or (B) IFNg (50 U/ml) for 48 hours. Western blot analysis was performed to probe for PD-L1 as well as STAT1/3 and their phosphorylated form (S727), which is required for the full transcriptional activity and biological function of STAT1/3. b actin was probed as a loading control. (A) N=3 or (B) N=2. A representative blot is shown.

**Supplementary Figure 6. gCTRL, MLH1 KO and MSH6 KO cells proliferate at the same rate.** Proliferation assay was carried out over ∼7 days using the Incucyte Live Cell Analysis system (Sartorius). N=1.

**Supplementary Figure 7. Silencing of SETD2 induces PD-L1 expression.** U2OS gCTRL, MLH1 KO and MSH6 KO cells were transfected with siRNA targeting SETD2 as well as a non-targeting control (siCTR). 24 hours later, cells were treated with IFNg for 72 hours. Whole cell lysates were extracted and analysed via western blotting to probe for PD-L1. Immunoblotting with antibodies against SETD2 confirmed the protein silencing. b actin was used as a loading control. N=3. A representative blot is shown.

## CRediT authorship contribution statement

**Kirsten Brooksbank:** Writing – review & editing, Data curation, Formal analysis, Investigation, Methodology, Writing – original draft. **Charlotte Smith:** Data curation, Formal analysis, Methodology, Writing – review & editing. **Eleni Maniati:** Writing – review & editing, Formal analysis. **Amy Gibson:** Writing – review & editing, Data curation, Investigation, Methodology. **Wai Yiu Tse:** Data curation, Formal analysis, Investigation, Methodology, Writing – review & editing. **Amy Kate Hall:** Data curation, Formal analysis, Investigation, Methodology, Writing – review & editing. **Jun Wang:** Writing – review & editing, Formal analysis. **Tyson V Sharp:** Conceptualization, Project administration, Supervision, Writing – review & editing. **Sarah A Martin:** Conceptualization, Formal analysis, Funding acquisition, Project administration, Resources, Supervision, Writing – original draft, Writing – review & editing.

## Declaration of competing interest

The authors declare that they have no known competing financial interests or personal relationships that could have appeared to influence the work reported in this paper.

## Appendix Supplementary materials

Image, image 1

Image, image 2

Image, image 3

Image, image 4

Image, image 5

Image, image 6

Image, image 7

Image, application 8
