# Supplementary figures and images for: The DNA mismatch repair protein, MSH6 is a novel regulator of PD-L1 expression
Source: Neoplasia. 2025 Jul 11;67:101207. doi: 10.1016/j.neo.2025.101207 (PMC12275031; doi:10.1016/j.neo.2025.101207)

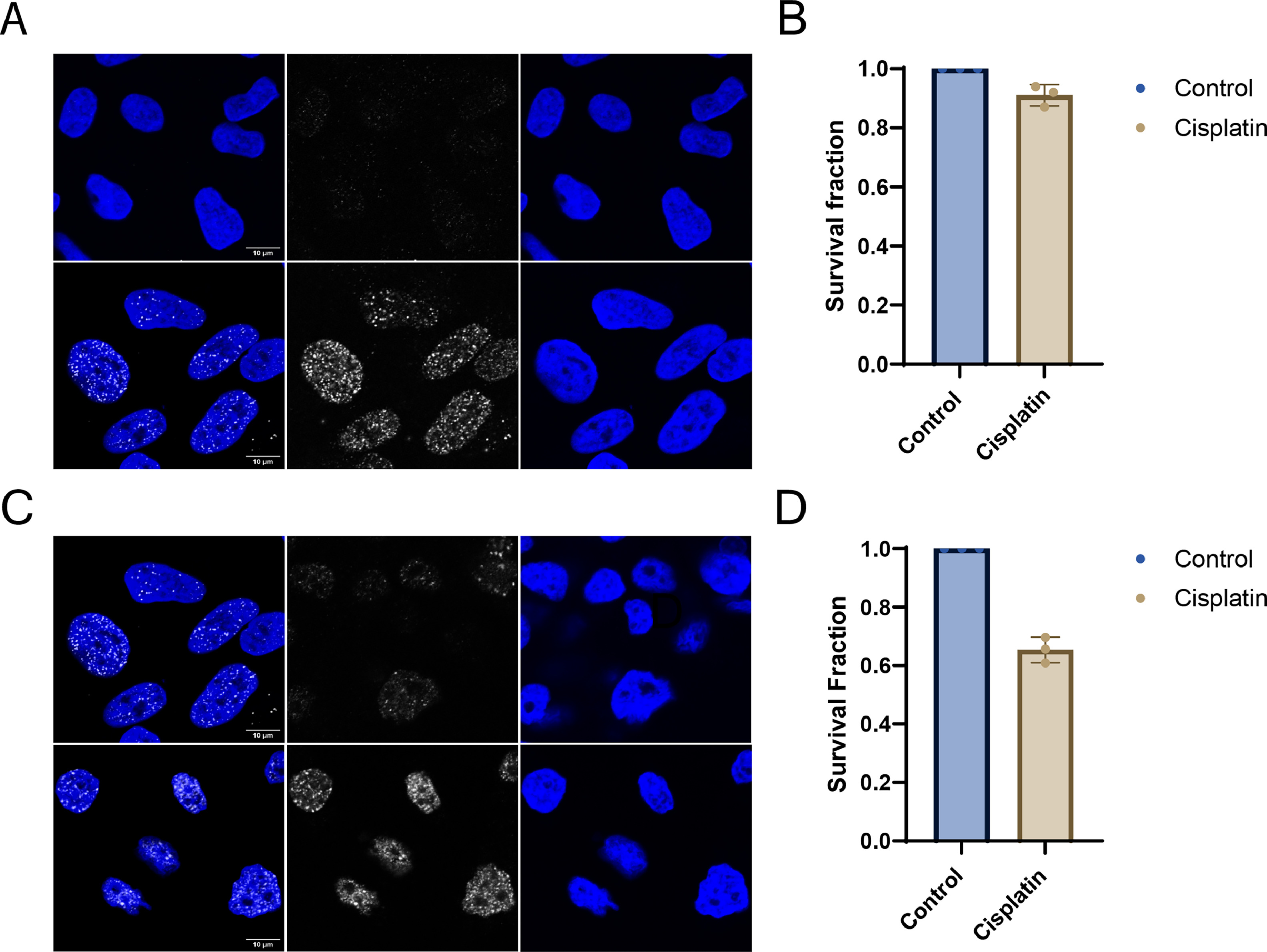

Supplement: Supplementary file 1 [file mmc1.jpg]

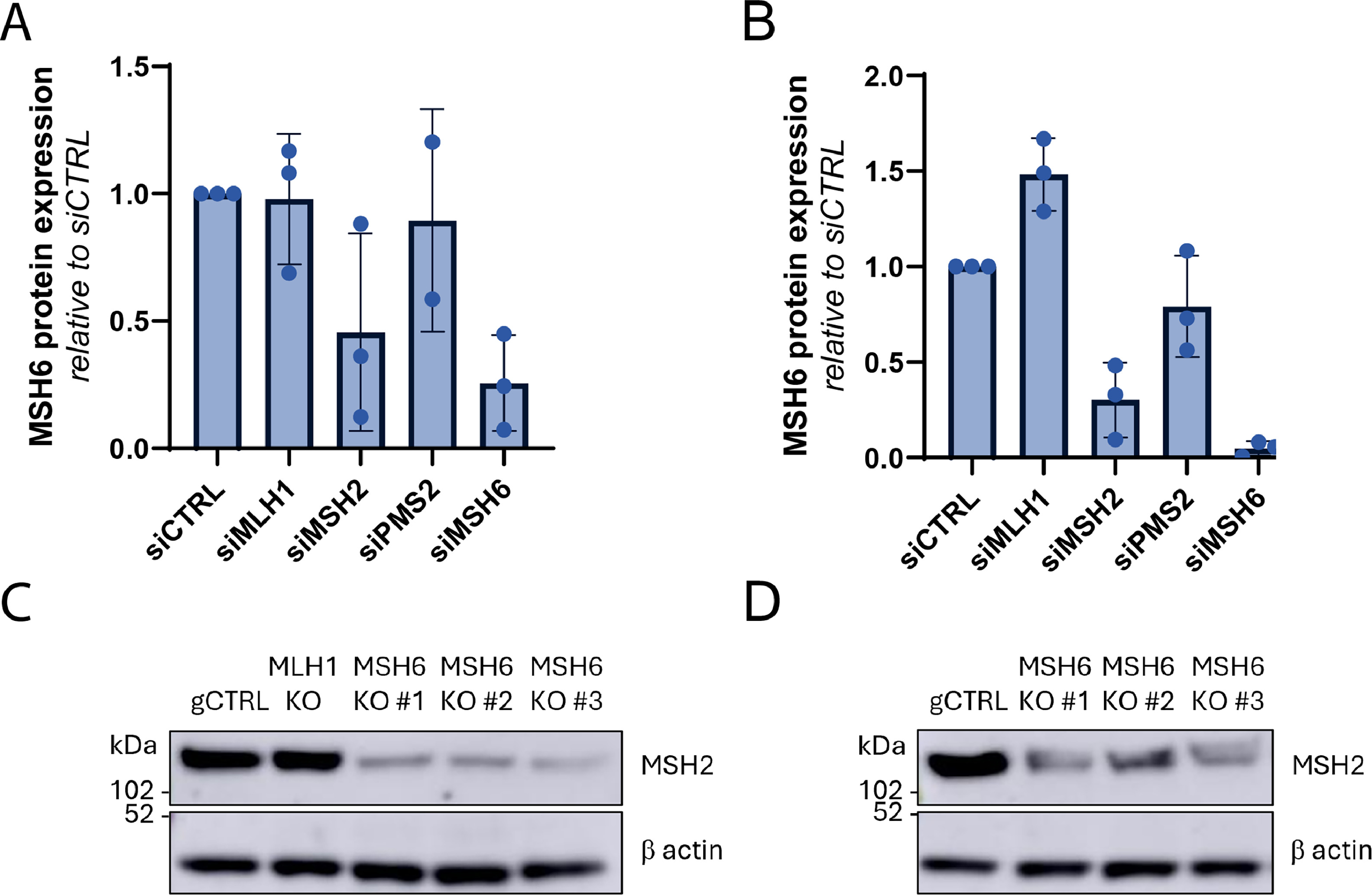

Supplement: Supplementary file 2 [file mmc2.jpg]

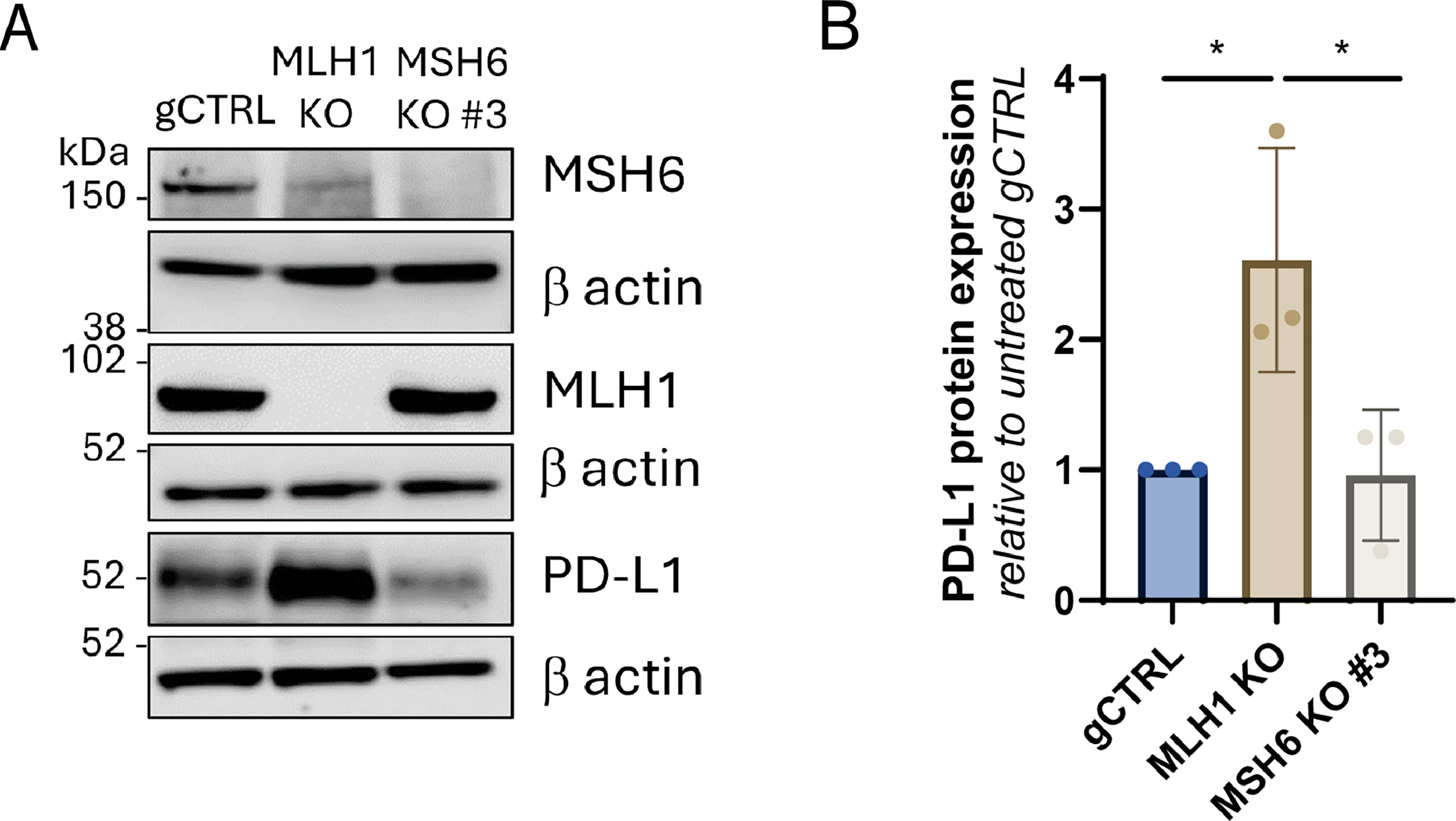

Supplement: Supplementary file 3 [file mmc3.jpg]

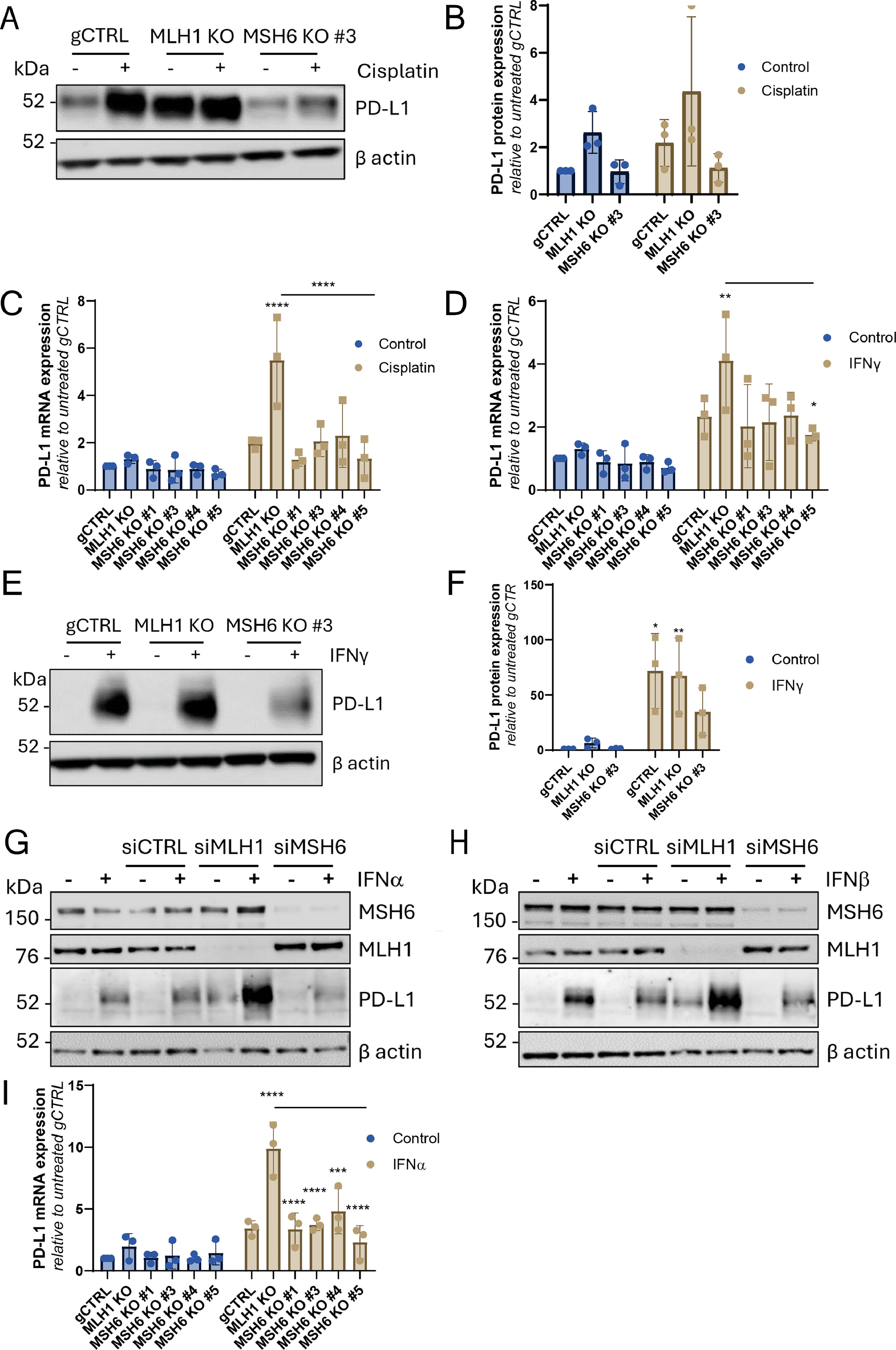

Supplement: Supplementary file 4 [file mmc4.jpg]

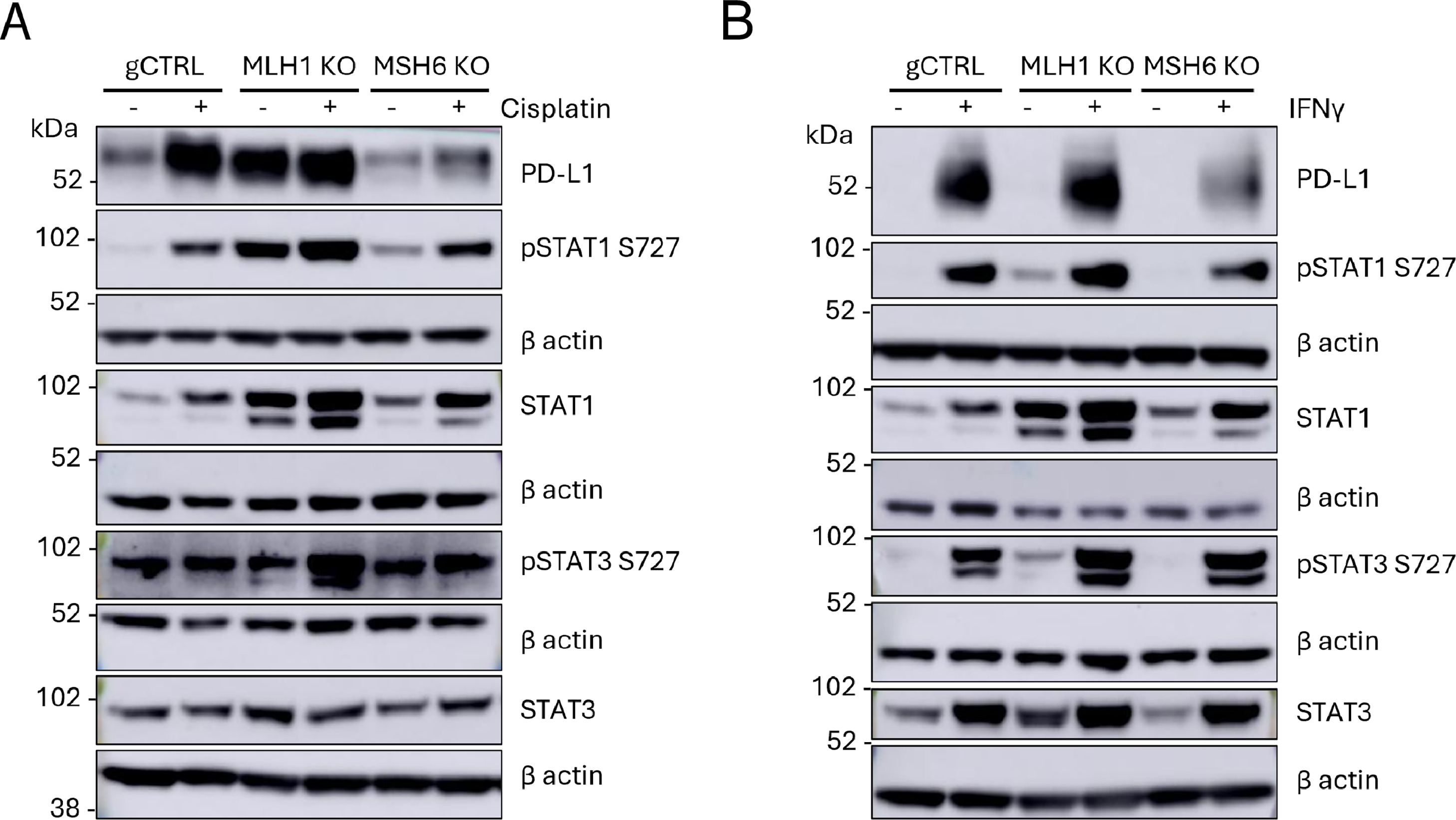

Supplement: Supplementary file 5 [file mmc5.jpg]

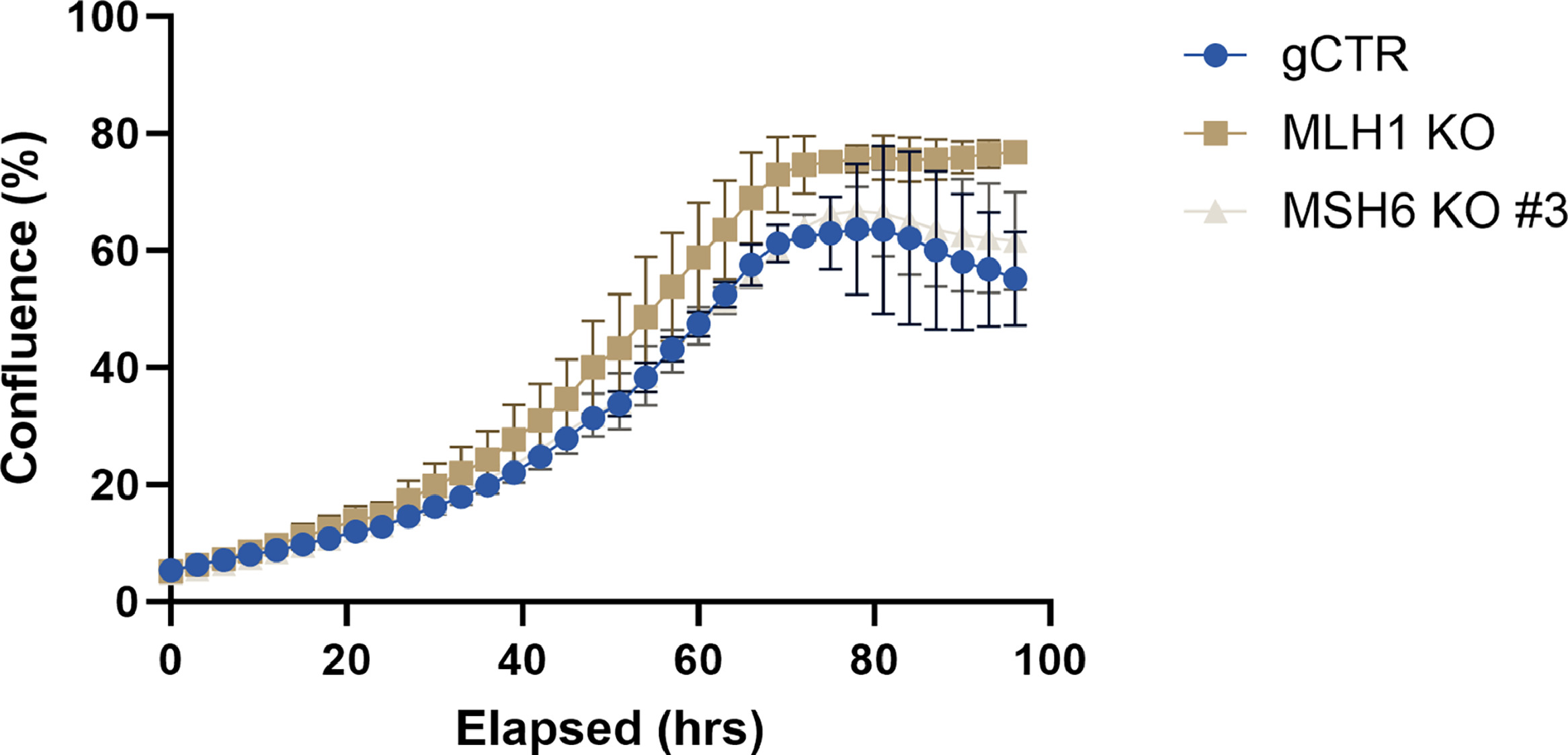

Supplement: Supplementary file 6 [file mmc6.jpg]

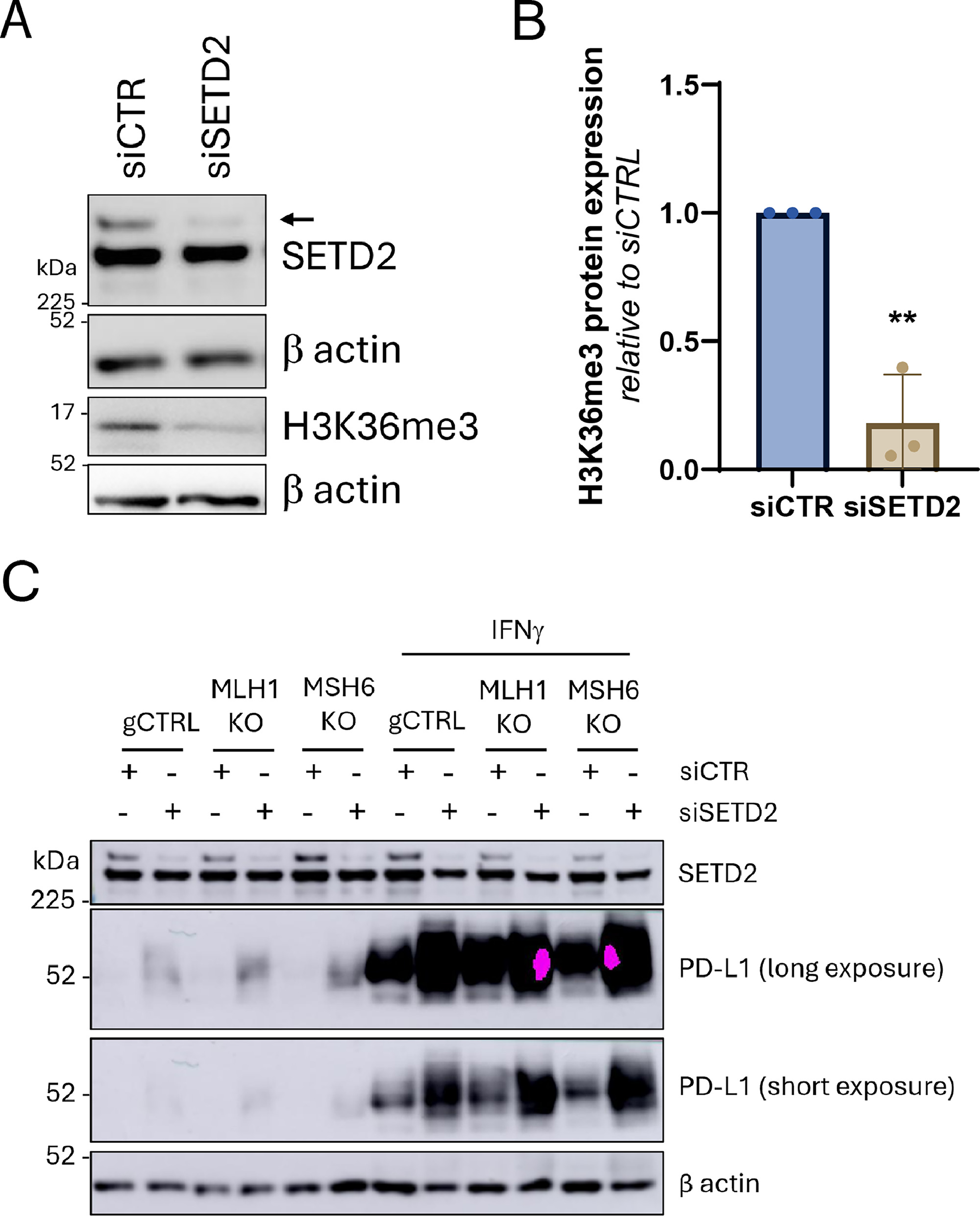

Supplement: Supplementary file 7 [file mmc7.jpg]
