# Supplementary material for: The DNA mismatch repair protein, MSH6 is a novel regulator of PD-L1 expression
Source: Neoplasia. 2025 Jul 11;67:101207. doi: 10.1016/j.neo.2025.101207 (PMC12275031; doi:10.1016/j.neo.2025.101207)
Supplement: Supplementary file 8 [file mmc8.docx]

Supplementary Table 1. Antibodies

| **Antibody** | **Manufacturer** | **Product code** |
| --- | --- | --- |
| ꞵ-tubulin | Cell signalling Technology | #2146 |
| ꞵ-actin | Cell signalling Technology | #4970 |
| MLH1 | Cell signalling Technology | #4256 |
| MSH2 | Cell signalling Technology | #2017 |
| PMS2 | Santa Cruz | sc-618 |
| MSH6 | Cell signalling Technology | #3995 |
| PD-L1 (human) | Cell signalling Technology | #13684 |
| PD-L1 (mouse) | Abcam | EPR20529 |
| STAT1 | Cell signalling Technology | #9172 |
| pSTAT1 | Cell signalling Technology | #9177 |
| STAT3 | Cell signalling Technology | #12640 |
| pSTAT3 | Cell signalling Technology | #9134 |
| SETD2 | Protein Tech | 55377-1-AP |
